# The relationship between head injury and the haemodynamic response to tracheal intubation

**DOI:** 10.1186/1757-7241-20-S1-O6

**Published:** 2012-03-22

**Authors:** DG Nevin, Z Perkins, DL Lockey

**Affiliations:** 1 Essex & Hertfordshire Air Ambulance Trust, Colchester, UK; 2 London’s Air Ambulance, Barts & The London Trust, London, UK; 3 Kent, Surrey & Sussex Air Ambulance Trust, Kent, UK; 4 Frenchay Hospital, North Bristol NHS Trust, Bristol, UK

## Introduction

Tracheal intubation is frequently required in the early management of head injured patients. By providing a secure airway and allowing controlled ventilation this intervention may reduce the incidence of secondary brain injury, a significant cause of morbidity and mortality. The procedure itself may, however, provoke a potentially harmful sympathetic response in head injured patients. Although reduced consciousness is thought to suppress airway reflexes, the influence of head injury on these reflexes is unknown. We aim to describe the influence of head injury severity on the haemodynamic response to laryngoscopy and tracheal intubation.

## Methods

We performed a retrospective analysis of 97 consecutive head injured patients who underwent tracheal intubation in a physician-led Helicopter Emergency Medical Service. Primary outcome was the acute haemodynamic response to the procedure. An adverse response was defined as a greater than 20% change from baseline recordings.

## Results

The incidence of an adverse hypertensive response to laryngoscopy and tracheal intubation was 80%. In 11% of cases blood pressure increased by ≥ 100%. There was attenuation in the heart rate (p=0.075), mean arterial pressure (p=0.066) and systolic blood pressure (p=0.024) response with increasing head injury severity. In the most severe head injuries (GCS 3), the incidence of an adverse hypertensive response was 63%.

## Conclusions

Head injury attenuates the haemodynamic response to laryngoscopy and tracheal intubation. This effect is unpredictable and insufficient to prevent an adverse haemodynamic response in even the most severe head injured patients. The need to attenuate the haemodynamic response should be assessed independently of head injury severity.

